# Adaptive Reproductive Strategies of an Ectoparasitoid *Sclerodermus guani* under the Stress of Its Entomopathogenic Fungus *Beauveria bassiana*

**DOI:** 10.3390/insects14040320

**Published:** 2023-03-27

**Authors:** Yun Wei, Li Li, Shumei Pan, Zhudong Liu, Jianting Fan, Ming Tang

**Affiliations:** 1 School of Life Sciences, Guizhou Normal University, Guiyang 550025, China; 2 Institute of Life Science, College of Life Science and Green Development, Hebei University, Baoding 071002, China; 3 National Joint Local Engineering Laboratory for High-Efficient Preparation of Biopesticide, School of Forestry and Biotechnology, Zhejiang A & F University, Hangzhou 311300, China; 4 Key Laboratory of State Forestry Administration on Biodiversity Conservation in Karst Mountain Area of Southwest of China, School of Life Science, Guizhou Normal University, Guiyang 550025, China

**Keywords:** *Sclerodermus guani*, *Beauveria bassiana*, reproduction, interspecific interactions, host-parasite interactions

## Abstract

**Simple Summary:**

This article studies the interaction between two parasites, a parasitoid (*Sclerodermus guani*) and an entomopathogenic fungus (*Beauveria bassiana*) on the same host, a longicorn beetle *Monochamus alternatus*. We focused on the survival and reproduction of the parasitoid’s parent and its offspring fitness under different concentrations of *B. bassiana* suspension in the laboratory. The results show that *S. guani* parent females carrying higher concentrations of the pathogen shorten the pre-reproductive time and regulate their own fertility and their offspring’s survival and development. Under the stress of the entomopathogenic fungus *B. bassiana*, the mortality effect of the parasitoid *S. guani* to its host *M. alternatus* was also assessed by the interspecific relationship model, which contained three dimensionless parameters: the ratio vulnerability, dilution ratio, and *P_R_* (the ratio of the total number of parasitoids successfully controlling host larvae *M. alternatus* to the total number of parasite events during parasitism). These findings shed light on the potential interspecific interactions between the two parasites which were able to coexist and communicate with their hosts in ecological contexts (with a high overlap in time and space) and cause interspecific competition and intraguild predation.

**Abstract:**

Complex interspecific relationships between parasites and their insect hosts involve multiple factors and are affected by their ecological and evolutionary context. A parasitoid *Sclerodermus guani* (Hymenoptera: Bethylidae) and an entomopathogenic fungus *Beauveria bassiana* (Hypocreales: Cordycipitaceae) shared the same host in nature, *Monochamus alternatus* (Coleoptera: Cerambycidae). They often encountered the semi-enclosed microhabitat of the host larvae or pupae. We tested the survival and reproduction of the parasitoid’s parent and its offspring fitness under different concentrations of *B. bassiana* suspension. The results show that *S. guani* parent females carrying higher concentrations of the pathogen shorten the pre-reproductive time and regulate their own fertility and their offspring’s survival and development. This minimal model of the interspecific interactions contains three dimensionless parameters, vulnerability (*θ*), dilution ratio (*δ*), and *P_R_*, which were used to evaluate the mortality effect of the parasitoid *S. guani* on its host *M. alternatus* under the stress of the entomopathogenic fungus *B. bassiana*. We compared the infection and lethal effect of the fungus *B. bassiana* with different concentrations to the parasitoid *S. guani* and the host larvae *M. alternatus*. At higher concentrations of the pathogen, the parasitoid parent females shorten the pre-reproductive time and regulate their own fertility and their offspring’s survival and development. At moderate concentrations of the pathogen, however, the ability of the parasitoid to exploit the host is more flexible and efficient, possibly reflecting the potential interspecific interactions between the two parasites which were able to coexist and communicate with their hosts in ecological contexts (with a high overlap in time and space) and cause interspecific competition and intraguild predation.

## 1. Introduction

**Figure A1:**
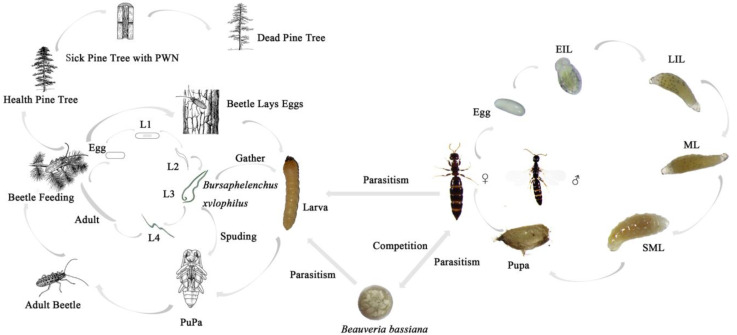
Interspecific relationships between the “*S. guani-B. bassiana-M. alternatus*”.

Natural enemy application is relatively safer and more environmentally friendly for controlling insect pests. However, the actual effect is affected by many ecological factors, especially various biotic factors in the common environment [1]. Most longicorn beetle larvae were able to encounter natural enemies in their pupal chamber on the trunks. There were complicated interspecific relationships, including parasitism, predation, competition, and even cooperation [2,3]. Interspecies relationships also determined the implementation effect and risk assessment of biological control [4,5]. Parasitoid species may rely on shared common resources (e.g., host species); thus, interactions among these parasitoid species can occur frequently (Appendix A: Figure A1) [6]. Both parasitoids and entomopathogenic fungus were often used to control the larvae and pupae of longicorn beetles and carpenter moths in hidden habitats [7,8], and the former can spread actively up to a certain distance, but the latter cannot. It can only spread passively through biotic and abiotic carriers or wind and rain. Parasitic wasps, such as *Sclerodermus guani* (Hymenoptera: Bethylidae), are just carriers that can travel long distances. Many studies show that *S. guani* female adults carrying pathogens were explored to prevent *Monochamus alternatus* (Coleoptera: Cerambycidae) [9], the vector beetle for the pinewood nematode that causes the destructive pine wilt disease [3,7]. They indirectly block the transmission of pine wood nematode *Bursaphelenchus xylophilus* (Aphelenchida: Aphelenchoididae), a major quarantine pest in the world [8,10].

*S. guani* is a gregarious ectoparasitoid (it oviposits on the surface of its host), good at drilling, searching, and attacking hosts in hidden habitats [11,12]. Once a suitable host is found, it will undergo stings paralysis, resulting in the complete death of the host. They will breed and raise their offspring until they leave [3,13,14]. During the parasitism process, parasitoids and *Beauveria bassiana* (Hypocreales: Cordycipitaceae) will meet in the host *M. alternatus*’s pupal chamber [15,16,17]. *B. bassiana* is a generalist entomopathogen due to the fact that it possesses a stereotypical pattern of pathogenicity genes towards many insect species [18]. Thus, the spores or hyphae of the pathogens may passively spread to the next host nest via the newly emerging adult offspring of the parasitoids. Several studies have reported this phenomenon in other insects and pathogens, e.g., *Phoridae* (Diptera: Phoridae) and *Trichogramma japonicum* (Hymenoptera: Trichogrammatidae).

The biological control technology of combining two natural enemies, *S. guani* and *B. bassiana*, has been conducted in some forest areas [2,15]. These approaches have gradually become a research hotspot in forest protection to find new and more efficient prevention methods [6,7,16,19]. Moreover, the evolution of insecticide resistance often shares the fundamental assumption that resistance is often associated with a fitness cost, which is crucial for understanding the population demographics of resistant insects, thereby managing the issue of resistance [20]. The broad spectrum of *B. bassiana* spores can infect various insects, including its hosts and their parasitic enemy insects [21]. To a large extent, the survival and reproduction of a parasitic wasp will be adversely affected by itself, carrying pathogenic spores of *B. bassiana* [17]. However, the notion of whether the evolution of insecticide resistance links to interspecific competition is currently poorly understood, especially in terms of the interaction between parasitoids, hosts, and pathogens.

Broad-spectrum pathogens are parasitic to the host and its parasitoids and develop faster. Once they kill and parasitize, the pathogen blocks its transmission carrier, and it is hard to leave the hidden semi-closed nest space [22,23,24]. Therefore, finding the mobility ride carrier, especially for those concealed biotypes, requires flexible trade-offs or coordination with the host. This study hypothesized that when *S. guani* carries the *B. bassiana* parasite host larvae of *M. alternatus*, the parasitoids will inhibit the growth process and develop pathogens by improving its parental reproduction and offspring survival. Under the stress of pathogens, the parasitoids will regulate their reproductive strategy and progeny developmental rate. The current experiments are based on earlier studies on the interactions between *S. guani*, *M. alternatus*, and *B. bassiana*. Three concentration gradients of *B. bassiana* (10^4^, 10^5^, and 10^6^ conidia mL^−1^) were used to examine the reproductive efficiency of parasitoids, as well as the survival, development, and transmission of their offspring. The present study aimed to evaluate the potential of the combined use of *S. guani* and *B. bassiana* to control *M. alternatus*. The first involves interactions between *S. guani* and *B. bassiana*, i.e., (a) the pathogenicity of *B. bassiana* to *S. guani* and (b) the pathogenic infection of immature offspring. The second involves interactions between *S. guani*, *B. bassiana*, and *M. alternatus*. The third utilizes the interspecific relationship model to evaluate interactions between parasitoids, hosts, and pathogens.

## 2. Materials and Methods

### 2.1. Insect Rearing

The experiments were conducted at the Pest Control and Resource Utilization Laboratory of Guizhou Normal University, Guiyang, China. The parasitoids of *S. guani* were provided by the Institute of Zoology, Chinese Academy of Sciences, and were raised by the Pest Control and Resource Utilization Laboratory of Guizhou Normal University for 48 successive generations [breeding was conducted parasitoid to host unit weight (1: 0.1 g), and placed in an incubator (25 °C, RH 65%, and 12 L:12 D), until the offspring emerged, and it was then transferred to a −8 °C refrigerator to refrigerate], and stable experimental populations were obtained. The larvae of *M. alternatus* were purchased in Kaili City, Guizhou Province (107.981° E, 26.566° N), and the larvae of *M. alternatusa* placed in a single-head single tube was installed in a test tube with sawdust (Sterilization and drying) and refrigerated at 4–5 °C. Before the experiment, the 3–4 instar larvae of *M. alternatus* were first washed with clean water and then disinfected with 10% alcohol. Finally, they were washed with distilled water and soaked with filter paper to absorb excess water from the surface of the larvae.

### 2.2. B. bassiana Suspensions

The strain of *B. bassiana* (GZUIFR-AS1) was provided by the Institute of Fungus Resources, Guizhou University. *B. bassiana* was seeded on PDA (Φ 90 mm) plates in a 25 °C dark for 14 days [25]. The conidia were harvested with a sterile spatula, suspended in sterile distilled water supplemented with 0.05% Tween 80 solution, and mixed well with a vortex mixer. To count the conidia directly, 10 μL was removed from the suspension with a pipette and repeated 3 times to obtain the mean. The final concentration was adjusted to 1 × 10^4^, 1 × 10^5^, and 1 × 10^6^ conidia mL^−1^ after dilution with sterile 0.05% Tween 80 solution, and sterile water was used as the control. Germination in conidial suspensions was assessed prior to experiments and was always kept above 95%.

### 2.3. Interactions between S. guani and B. bassiana

#### 2.3.1. The Pathogenicity of *B. bassiana* to *S. guani*

For the initial pathogen load of *S. guani* adult females, female adult parasitoids of *S. guani* were placed in a 120 mm Petri dish, and each female adult parasitoid was dripped with 0.1 mL of spore suspension of different concentrations (10^4^, 10^5^, and 10^6^ conidia mL^−1^), and allowed to fully crawl until the body was covered with the *B. bassiana* and then naturally air-dried. After setting 3 replicates per concentration, 60 test parasitoids per replicate were placed in an incubator (25 °C, RH = 65%, photoperiod 12 L:12 D) for 5 min, 1 d, 2 d, 3 d, 4 d, 5 d, and 6 d, and 20 vigorous test parasitoids were randomly selected and eluted with 0.05% Tween 80 solution. The effective number of *B. bassiana* carried by each female adult parasitoid was then calculated by the blood cell counting board (XB-K-25) and converted to the spore-carrying amount per parasitoid, and the process was repeated 3 times for each concentration.

For infestation, the dipping method was used, and the parent female adult parasitoids were infected in *B. bassiana* suspensions at 10^4^, 10^5^, and 10^6^ conidia mL^−1^ for 5 s. The filter paper absorbed the excess fungal fluid and transferred it into a clean and sterile Petri dish (Φ 90 mm). Five replicates were performed at each concentration, one female adult parasitoid was repeated for each replicate, and the infection of female adult parasitoid and the growth of hyphae were recorded every 24 h.

Based on the fertility of parental female adult parasitoids, the healthy larvae of *M. alternatus* larvae weighing 0.35–0.45 g were selected, sterilized, air-dried, weighed, and then placed in a clean glass test tube (Φ 75 mm). The parasitoids were placed in an incubator at 25 °C, and the ratio of test parasitoids to host unit weight (1: 0.1 g) was adopted to paralyze the host. Then, 30 replicates per concentration was set, and sterile water was used as the control. The activity ability and physiological state of the female parasitoids were observed and recorded every 24 h, and the adult female parasitoid survival rates (%), the lethality of the *S. guani* to *M. alternatus* pre-oviposition (d), oviposition duration (d), the longevity of *S. guani* (d), and the number of eggs laid (eggs/clutch; clutch is defined as the number of eggs released during a single spawning event) of each group of female parasitoids were recorded until death.

#### 2.3.2. The Pathogenicity of *B. bassiana* Infects the Immature Offspring

Immature offspring were divided into 6 time periods: the egg of *S.guani* (ES), early instar larva (EIL), late instar larva (LIL), mature larva (ML), spinning mature larva (SML), and pupa cocoon (PC) [14]. The egg of *S. guani* (ES), early instar larva (EIL), late instar larva (LIL), mature larva (ML), spinning mature larva (SML), and pupa cocoon (PC) of parasitoids were separated from the host surface with a fine bristle brush and placed in a Petri dish. The spore suspension of the 3 concentration gradients was prepared according to Section 2.2, and the dipping method was used. The eggs, early larvae, late larvae, and mature larvae were infected in 3 suspensions of *B. bassiana* at different concentrations of 5 s. The filter paper absorbed excess spore suspension. The larvae were placed into a sterile dish (Φ 90 mm). Each concentration was performed in 5 replicates, with one parasitoid of an egg or a larva per replicate. Photographs were taken with a Stereo Microscope (OLYMPUS SZX7, made in Japan) every 24 h to record the infection of *B. bassiana* in eggs or larvae. The conditions for cultivation are the same as those of the *M. alternatus* larvae.

### 2.4. Interactions between S. guani, B. bassiana, and M. alternatus

#### 2.4.1. The Pathogenicity of *B. bassiana* to *M. alternatus*

The treatment of 3–4 instar larvae of *M. alternatus* with different concentrations of *B. bassiana* suspension (10^4^, 10^5^, and 10^6^ conidia mL^−1^) was usde by the dipping method. The larvae of *M. alternatus* were placed separately into the spore suspension of different concentrations for 5 s, quickly taken out and placed in a 10 mL finger-shaped tube, fed with sterilized and dried sawdust, and controlled with 3–4 instar larvae of *M. alternatus* soaked with 0.05% Tween 80 solution [26]. The treated larvae were incubated at a constant temperature of 25 °C in an incubator, with 20 replicates per concentration. The mortality of the larvae was noted and recorded at 24 h intervals. Dead larvae were kept at 25 °C for 15 days to evaluate the pathogenicity in *M. alternatus*.

#### 2.4.2. The Model

Mesterton-Gibbons and Hardy [27] developed a model which can be applied to mammalian predators that attempt to capture and subdue large and dangerous prey. Moreover, this model can be applied to female parasitoids that attack and, if successful, paralyze aggressive hosts, providing the only feeding resource for their offspring. These mammals or parasitoids risk death and aggression when interacting with large prey. Parasitoids actively search for, locate, and use the host and the environment. Once a suitable host is located, sting paralysis entirely kills the host, but parasitoids also risk dying or being attacked as they control their host. So, how do parasitoids cope with reducing this risk?

#### 2.4.3. The Mortality Effect of *S. guani* on the Host under *B. bassiana* Stress

We used biological factors to investigate the risks faced by parasitoids and the time required to subdue the host under different concentrations of *B. bassiana* stress, which determines whether the parent can provide fully adequate food for the offspring, and sterile water was used as the control. In the research system of “parasitoids-host-pathogens”, a new model is proposed to evaluate the LE (lethal effect: the effect on survival) [28] and PE (parasitic effect: the average number of parasitic pests per natural enemy) [29] of *S. guani* on hosts under pathogenic fungal stress using *D*, *S*, *B*, *t*, *θ*, *δ*, and *P_R_*. Parasitoids overcome their hosts in one of two ways: the parasitoids either kill the host or the parasitoids are bitten to death by the host. Parasitoids’ fitness in the first instance is 1 and their fitness in the second instance is 0:

(1) $$\theta =\frac{S+t}{S}$$

(2) $$P_{R}=\frac{S}{2S+t}$$

(3) $$\delta =\frac{S+3\left(S+t\right)}{4\left(S+t\right)}$$

*D*(d): the pre-oviposition of female adult parasitoids;
*S*(d): the female adult parasitoids completely paralyze the host at the time;
*B*(d): the time when the female adult parasitoid delivers a fatal blow to the host;
*t*(d): the female adult parasitoids die after the host of the duration of egg laying;
*θ*: the ratio of the time it takes for the female adult parasitoids to completely paralyze the host during the pre-oviposition of female adult parasitoids;
*P_R_*: the parasitic probability of *S. guani* after paralyzing host *M. alternatus* larvae;
*δ*: the antagonistic effect of *S. guani* and *B. bassiana* (the greater the antagonism, the worse the parasitoid, the weaker the lethal host effect).

### 2.5. Date Analysis

We used SPSS 26.0 statistical software for data analysis and archiannelid percentage data transformation before analysis. The Kolmogorov–Smirnov test was then used to determine whether the data follow a normal distribution. The data were subjected to analyses of variance and the means were compared using TukeyÕs test, with significance determined at *p* < 0.05. Photoshop CS6 and Origin 2021 were used to make drawings.

## 3. Results

### 3.1. The Pathogenicity of B. bassiana to S. guani

**Figure 1:**
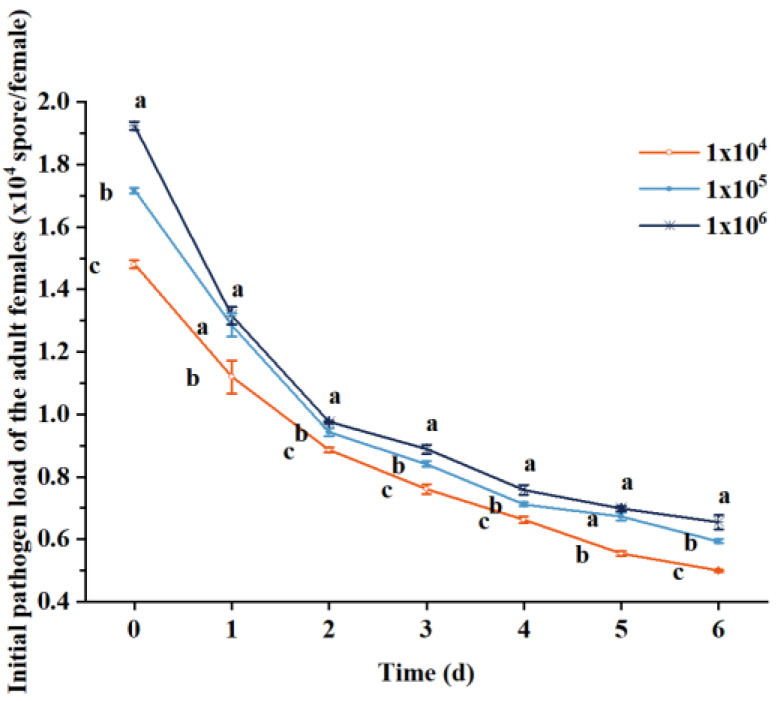
Initial pathogen load of the female adults of *S. guani* under different concentrations of *B. bassiana* spore suspensions. Different letters above the bars indicate significant differences (mean ± SE, *n* = 3 in each treatment).

The daily variation in the spore-carrying capacity of female adult parasitoids to various concentration suspensions showed that there were significant differences in the initial spore-carrying capacity of female adult parasitoids to various concentration suspensions at three concentrations: 0 d (F_2,1_ = 349.077, *p* < 0.01), 1 d (F_2,1_ = 10.842, *p* < 0.05), 2 d (F_2,1_ = 25.992, *p* < 0.01), 3 d (F_2,1_ = 26.726, *p* < 0.01), 4 d (F_2,1_ = 18.816, *p* < 0.01), 5 d (F_2,1_ = 59.546, *p* < 0.01), and 6 d (F_2,1_ = 29.701, *p* < 0.01), and the initial carrying capacities from large to small were 10^6^ *>* 10^5^ *>* 10^4^ conidia mL^−1^ (Figure 1). The overall trend shows that the higher the concentration of *B.bassiana* carried by female adult parasitoids, the higher the amount of spore shedding.

**Figure 2:**
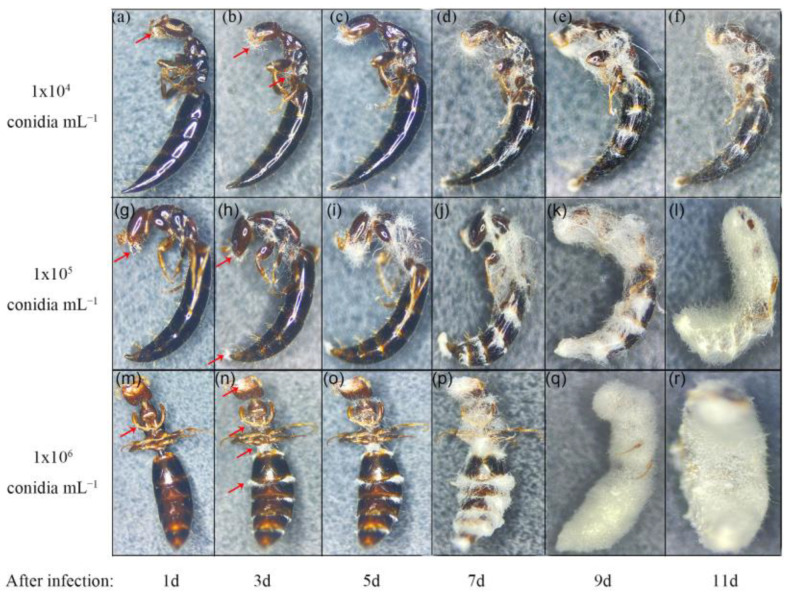
Infestation of pathogens against parental female adults of *S. guani* under different concentrations of *B. bassiana* spore suspensions (red arrow indicates the dead larvae) (a–f: growth of hyphae on the body surface of *S. guani* at 10^4^ conidia mL^−1^ *B. bassiana* suspension; g–l: growth of hyphae on the body surface of *S. guani* at 10^5^ conidia mL^−1^ *B. bassiana* suspension; m–r: growth of hyphae on the body surface of *S. guani* at 10^6^ conidia mL^−1^ *B. bassiana* suspension).

Before inoculation, the body surface of the parasitoid was black. On the 1st day after inoculation, the parasitoid crawled normally; on the 3rd day after inoculation, with the increase in concentration, the vitality of the parasitoid became weaker and weaker until it died. On the 5th day after inoculation, the parasitoid died and hyphae first grew from the mouthparts and appendages of parasitoids at various concentrations. On the 7th day after inoculation, the hyphae growth rate was 10^6^ *>* 10^5^ *>* 10^4^ conidia mL^−1^. On the 9th day after inoculation, at 10^5^ and 10^6^ conidia mL^−1^, a large number of hyphae grew from the larvae body, while at 10^4^ conidia mL^−1^, only the hyphae around the mouthparts germinated. On the 11th day after inoculation, the parasitoid was completely wrapped by the hyphae and produced spores at 10^6^ conidia mL^−1^, while the parasitoid was half-wrapped by the hyphae at 10^5^ conidia mL^−1^. At this time, the hyphae were only beginning to grow in large numbers at 10^4^ conidia mL^−1^ (Figure 2).

**Figure 3:**
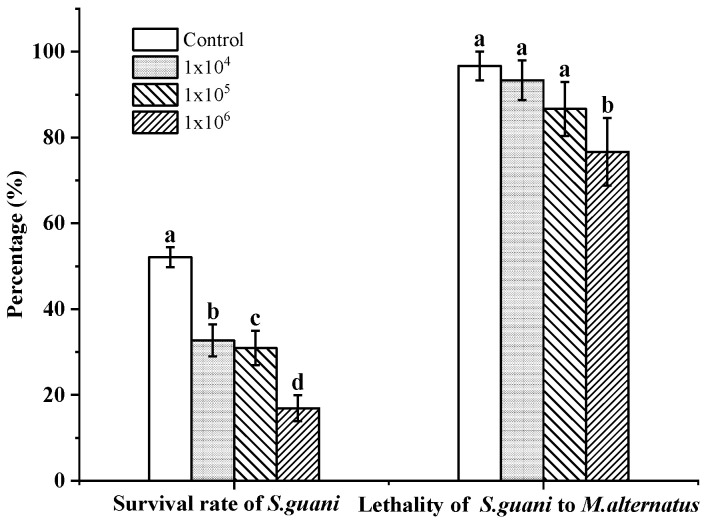
The survival rate of *S. guani* and lethality of *S. guani* to *M. alternatus* at different concentrations of *B. bassiana* spore suspension. Different letters above the bars indicate significant differences (mean ± SE, *n* = 30 in each treatment, *p* < 0.05).

The survival rate of *S. guani* (F_3,116_ = 3.509, *p* < 0.05) and lethality of *S. guani* in their hosts (F_3,116_ = 16.012, *p* < 0.01) treated with different concentrations were lower than those of the control (Figure 3). As the concentration increased, the survival rate of *S. guani* and lethality of *S. guani* in their hosts were 10^4^ conidia mL^−1^ (32.72% ± 3.735; 93.33% ± 4.63), 10^5^ conidia mL^−1^ (30.95% ± 4.03%; 86.67% ± 6.31), and 10^6^ conidia mL^−1^ (16.94% ± 3.05; 76.67% ± 7.85). Compared with the control, the survival rate of *S. guani* decreased by 19.39%, 21.16%, and 35.17%, respectively, while the lethality of *S. guani* to their hosts decreased by 3.34%, 10%, and 20%, respectively.

**Figure 4:**
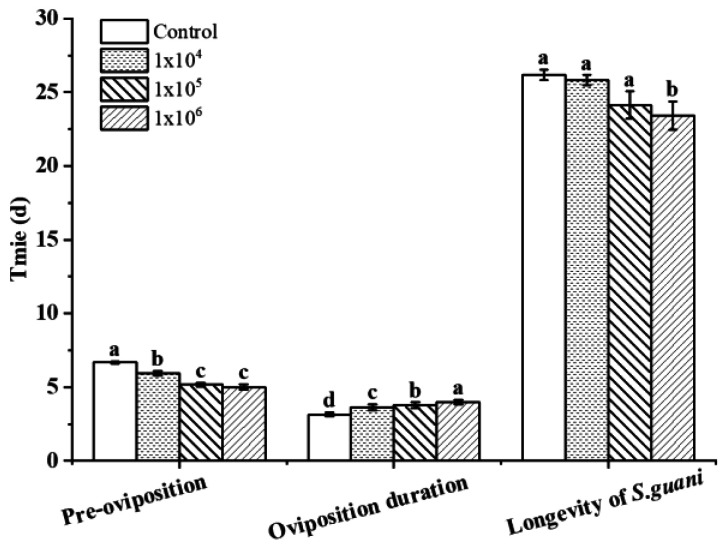
Performance of *S. guani* under different concentrations of *B. bassiana* spore suspensions: pre-oviposition, Oviposition duration, and Longevity of *S. guani* under different concentrations of *B. bassiana* spore suspension. Different letters above the bars indicate significant differences (Mean ± SE, *n* = 30 in each treatment, *p* < 0.05).

Under different concentrations of treatment, the pre-oviposition (F_3,116_ = 97.669, *p* < 0.01) was less than the control and the oviposition duration (F_3,116_ = 44.422, *p* < 0.01) was higher than the control. Pre-oviposition and oviposition duration of *S. guani* was significantly different. Although the longevity of *S. guani* (F_3,116_ = 3.509, *p* < 0.05) in various treatment was lower than the control, the longevity was statistically different only when it was treated with *B. bassiana* at 10^6^ conidia mL^−1^ (F_3,116_ = 3.509, *p* < 0.05). As the concentration increased, the pre-oviposition, oviposition duration, and longevity of *S. guani* at various concentrations were: 10^4^ conidia mL^−1^ (5.93 ± 0.09 d; 3.63 ± 0.09 d; 25.83 ± 0.75 d), 10^5^ conidia mL^−1^ (5.17 ± 0.11 d; 3.77 ± 0.09 d; 24.12 ± 0.85 d), and 10^6^ conidia mL^−1^ (5.00 ± 0.18 d; 4.00 ± 0.19 d; 23.41 ± 0.68 d) (Figure 4). Compared with the control, the pre-oviposition of *S. guani* was shortened by 0.74 d, 1.50 d, and 1.67 d, respectively, and the oviposition duration of *S. guani* was prolonged by 0.63 d, 0.77 d, and 1.00 d, respectively. The longevity of *S. guani* was shortened by 0.57 d, 2.22 d, and 2.93 d, respectively (Figure 4).

**Figure 5:**
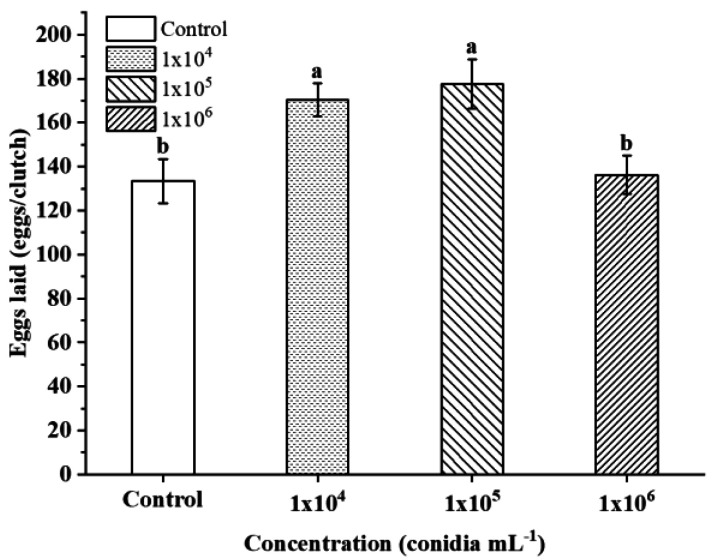
Eggs laid off *S. guani* under different concentrations of *B. bassiana* spore suspension. Different letters above the bars indicate significant differences (mean ± SE, *n* = 30 in each treatment, *p* < 0.05).

Under different concentrations, only 10^4^ conidia mL^−1^ (170.27 ± 9.149) eggs/clutch and 10^5^ conidia mL^−1^ (177.5 ± 11.167) eggs/clutch of *S. guani* were significantly different from those of the control (133.37 ± 6.765) eggs/clutch (*p* < 0.05). Compared with the control, the number of eggs laid at 10^4^ and 10^5^ conidia mL^−1^ increased by 36.9 and 44.13 grain, respectively; however, the egg laid of 10^6^ conidia mL^−1^ (136.20 ± 9.863) eggs/clutch was similar to that of the control, with no significant difference (*p >* 0.05) (Figure 5).

### 3.2. Pathogens Infect Immature Offspring

**Figure 6:**
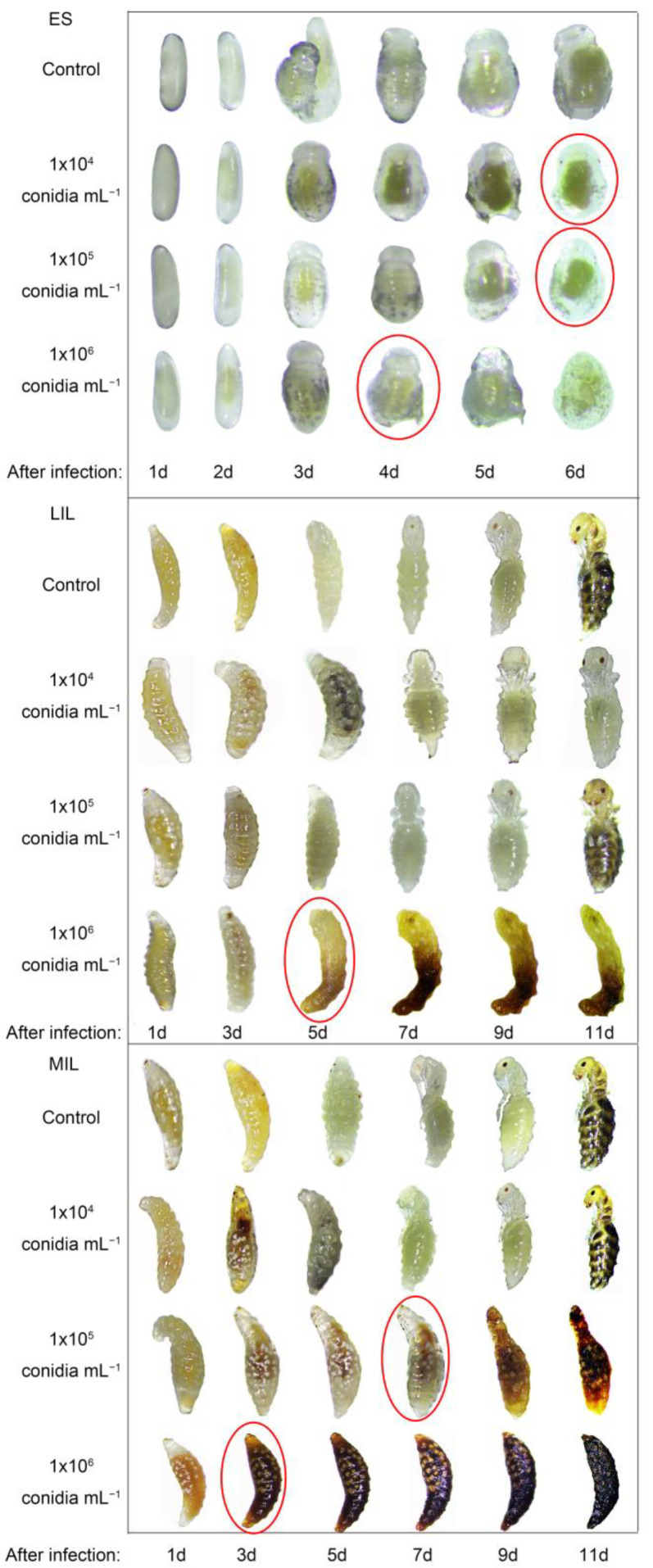
Infestation of pathogens against offspring of *S. guani* under different concentrations of *B. bassiana* spore suspensions. The red circle indicates that the larva has died. ES: egg of *S. guani*; LIL: late instar larval; ML: mature larval.

The infection of immature offspring of *S. guani* at each developmental stage is shown in Figure 6. Under different concentrations, the death rate of immature offspring of *S. guani* was 10^6^ *>* 10^5^ *>* 10^4^ conidia mL^−1^.

**Figure 7:**
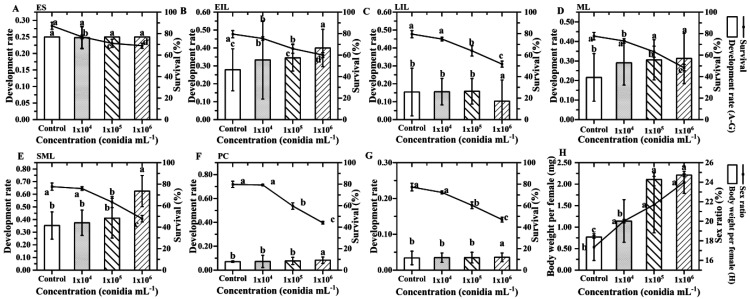
The performance of immature *offspring* of *S. guani* under different concentrations of *B. bassiana* spore suspensions. The mean development rate and survival rate of immature *offspring* of *S. guani*. (**A**) ES: egg of *S. guani*; (**B**) EIL: early instar larval; (**C**) LIL: late instar larval; (**D**) ML: mature larval; (**E**) SML: spanning mature larval; (**F**) PC: pupa cocoon; (**G**) whole generation and survival of the offspring of *S. guani*; (**H**) sex ratio and body weight per female of the offspring of *S. guani*. Different letters above the bars indicate significant differences (mean ± SE, *n* = 30 in each treatment, *p* < 0.05).

After inoculation with different concentrations of *B. bassiana* suspension, the development rate of the offspring larvae increased, the survival rate decreased, and the body weight per female and male ratio of the offspring of *S. guani* increased. Compared with the control, the development rate of ES (F_3,116_ = 1, *p >* 0.05) (Figure 7A) was not affected by the concentration of spore suspension, while the development rates of EIL (F_3,116_ = 10.116, *p* < 0.01) (Figure 7B), LIL (F_3,116_ = 20.022, *p* < 0.01) (Figure 7C), ML (F_3,116_ = 13.622, *p* < 0.01) (Figure 7D), SML (F_3,116_ = 19.526, *p* < 0.01) (Figure 7E), and PC (F_3,116_ = 17.577, *p* < 0.01) (Figure 7F) were all accelerated. Compared with the control, the average developmental duration of the larvae of offspring 10^4^, 10^5^, and 10^6^ conidia mL^−1^ (F_3,116_ = 8.432, *p* < 0.01) was shortened by 2.43 d, 3.68 d, and 6.50 d, respectively. Compared with control, the survival rates of larvae in different insect states decreased for ES (F_3,116_ = 8.362, *p* < 0.01) (Figure 7A), EIL (F_3,116_ = 8.454, *p* < 0.01) (Figure 7B), LIL (F_3,116_ = 13.907, *p* < 0.01) (Figure 7C), ML (F_3,116_ = 13.095, *p* < 0.01) (Figure 7D), SML (F_3,116_ = 15.288, *p* < 0.01) (Figure 7E), and PC (F_3,116_ = 22.324, *p* < 0.01) (Figure 7F). The average survival rate of the offspring larvae of 10^4^, 10^5^, and 10^6^ conidia mL^−1^ decreased by 4.39%, 15.90%, and 27.28%, respectively. As the concentration increased, the sex ratio (F_3,116_ = 7.112, *p* < 0.01) and the body weight per female (F_3,116_ = 10.158, *p* < 0.01) of the offspring parasitoids increased. Compared with the control, the male sex ratio of parasitoids from the offspring of 10^4^, 10^5^, and 10^6^ conidia mL^−1^ increased by 2.68%, 4.36%, and 6.64%, respectively (Figure 7H). The body weight per female increased by 0.50, 1.32, and 1.50 mg, respectively (Figure 7H).

### 3.3. Pathogens Infect M. alternatus

**Figure 8:**
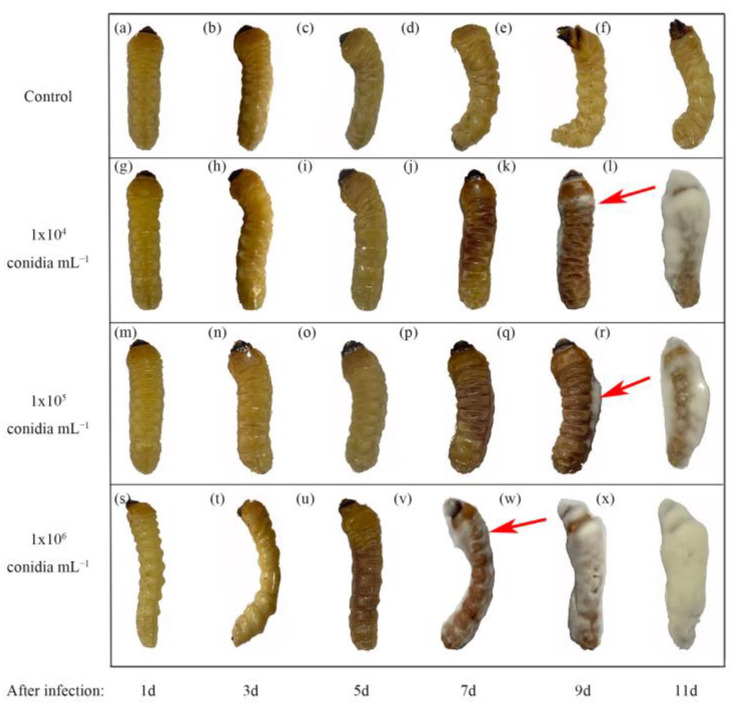
The infection of *M. alternatus* larvae under different concentrations of *B. bassiana* spore suspensions (the red arrow indicates the growth of *B. bassiana* hyphae on the surface of *M. alternatus* larvae) (a–f: growth of hyphae on the body surface of *M. alternatus* larvae at the control; g–l: growth of hyphae on the body surface of *M. alternatus* larvae at 10^4^ conidia mL^−1^ *B. bassiana* suspension; m–r: growth of hyphae on the body surface of *M. alternatus* larvae at 10^5^ conidia mL^−1^ *B. bassiana* suspension; s–x: growth of hyphae on the body surface of M. alternatus larvae at 10^6^ conidia mL^−1^ *B. bassiana* suspension).

After inoculation with different concentrations (10^4^, 10^5^, and 10^6^ conidia mL^−1^) of *B. bassiana* spore suspension, on the 1st day, the larvae typically twitched and displayed strong vitality. On the 3rd day after inoculation, the larvae moved slowly. On the 5th day after inoculation, the color of larval epidermis changed to purple and gradually deepened to brown at 10^6^ conidia mL^−1^. On the 7th day after inoculation, the larva began to grow hyphae on its surface at 10^6^ conidia mL^−1^, and the larvae died at 10^4^ and 10^5^ conidia mL^−1^. On the 9th day after inoculation, the body of the zombie insect was wrapped in hyphae at 10^6^ conidia mL^−1^, and a small amount of hyphae appeared in the body of the zombie insect at 10^4^ and 10^5^ conidia mL^−1^. On the 11th day after inoculation, the zombie insect was completely wrapped by the hyphae at 10^4^, 10^5^, and 10^6^ conidia mL^−1^ (Figure 8).

### 3.4. Mortality Effect of S. guani on the Host under B. bassiana Stress

**Figure 9:**
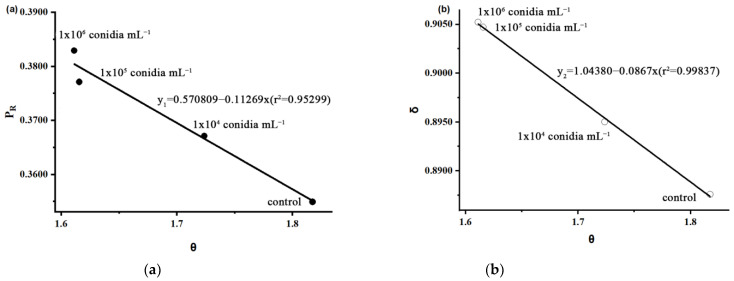
The lethal effect and parasitic effect of *S. guani* on the larvae of *M. alternatus* under different concentrations of *B. bassiana* spore suspension: (**a**) time-lethal effect; (**b**) time antagonism.

Given the concentration threshold in this experiment (10^4^–10^6^ conidia mL^−1^), according to the linear regression equations y_1_ = 0.57809 − 0.12269x (r^2^ = 0.95299) and y_2_ = 1.04308 − 0.0867x (r^2^ = 0.99837) which show that the value of θ decreased, P_R_ and δ increased as the concentration increased (Table 1). This indicates that the antagonism between *B. bassiana* and *S. guani* increases. It takes less time for *S. guani* to kill and paralyze their hosts, but uniform hosts are less efficient. *S. guani* will shorten the duration of the host’s paralysis and a deadly period when confronted with *B. bassiana*, and will prevent their growth for the better use of resources provided by the host in the face of a large host prey (Figure 9).

## 4. Discussion

The exposure interval between the host and the parasitoids is an important variable in the interaction efficiency of applying a particular entomopathogenic fungus, which could create neutral, positive, or negative relationships [18]. To resist microbial invasion, various animal species have thus evolved diverse means to prevent and combat the detrimental effects of microbial competitors and pathogens on their offspring [30]. Our work shows that *S. guani* parent females carrying higher concentrations of the pathogen shorten pre-oviposition and regulate their own fertility and their offspring’s survival and development. At moderate concentrations of the pathogen, however, the ability of the parasitoids to exploit the host is more flexible and efficient, possibly reflecting the potential interspecific interactions between the two parasites which were able to coexist and communicate with their hosts in ecological contexts (with a high overlap in time and space) and cause interspecific competition and intraguild predation.

The survival rate and reproductive efficiency of the parental female adults of *S. guani* were significantly reduced, and the host lethality was low, but the development time was short of the offspring of *S. guani* at 10^6^ conidia mL^−1^. Females had long longevity, high reproductive efficiency, a high survival rate, host lethality of offspring parasitoids, and long offspring development time at 10^4^ conidia mL^−1^. Although eggs laid were maximum, female adult longevity, reproductive effects, offspring survival, and host lethality were notably lower at the optimum concentration than at 10^4^ conidia mL^−1^. *S. guani* reduced eggs laid, resulting in different offspring densities and affecting offspring weight at 10^6^ conidia mL^−1^. This is because those female parasitoids need to invest and nurture in reproduction and breeding, tending to ensure better survival among offspring [31]. Cultivating a stable number of female offspring is conducive to the reproduction and development of the whole population [3,7]. The quality of individual host mortality rates is lower than that of parental female offspring because of *B. bassiana* mortality and the after-effect of parasitoids. This is because female parents are willing to take precautions to avoid the risk of infection as their offspring have low activity levels, and their infection increases the chance of death [32]. However, after carrying *B. bassiana*, the parent of *S. guani* should also avoid the passive influence of the pathogen on its offspring. Parents may shorten the pre-oviposition, extend the oviposition duration, and shorten the average development duration of the offspring to cope with the infection of the spore suspension.

Multiple natural enemies often coexist and communicate with their hosts in ecological contexts (with a high overlap in time and space) and cause interspecific competition and intraguild predation (IGP) [32,33]. For parasitoids, the host serves as a vital source of food for their offspring, and the pupal chamber of the host serves as a suitable shelter. When the parasitoids sting and paralyze the host, it will be actively attacked and hurt by the host [3,6,33], leading to behavioral aggression, chemical and physical defense, and increased risk intensity with host size and developmental progress [34,35]. Parasitoids need to identify these imminent risks and balance the contradiction between mortality risk, resource utilization efficiency, and the maximum possibility of population reproduction [27,36]. Liu used 1–2 parasitoids to the host *M. alternatus* to assess resistance and survival based on the risk of death, parasitic success, and the “parasitoid-host-pathogen” interaction described [3]. The lethal effect of parasitoids on the host was determined by *P_R_*, vulnerability (*θ*), and the dilution ratio (*δ*). Under the treatment of 10^4^, 10^5^, and 10^6^ conidia mL^−1^, the lethal effect of parasitoids on hosts decreased with the increase in *θ*, and the time of subduing hosts was shortened with the increase in *θ*. These results show that under certain stress of the pathogenic fungus, the parasitoids will encounter competition, infection, and death threats. Parasitoids thus accelerated the process of capturing the host to reduce the minimum impact of pathogenic fungus on itself.

When insect pathogenic fungus and natural enemies (predatory and parasitic natural enemies) coexist in the same ecosystem, they will interact at the trophic level, which will happen to IGP [37]. Paralyzing the host is a vital stage for parasitoids in effective parasitism and reproduction, but they risk dying because of the host’s defenses [3]. In the semi-enclosed pupal chamber, *B. bassiana* competed with *S. guani* in parasitism and reproduction. By infecting the host, *B. bassiana* could indirectly affect the survival and reproduction of parasitoids [17]. Furthermore, the entomopathogen can compromise the quality of the host (nutritional or physiological alterations) [18]. So, the rapid growth of *B. bassiana* affects the state of the host, thus affecting the parasitoid’s survival, oviposition, growth, and development [12,15]. In this case, parasitoids may actively take care to avoid the adverse effects of *B. bassiana*. The study found that the offspring without parental care were more susceptible to *B. bassiana* infection than those with parental care [30]. This experiment confirmed that stress under 10^5^ conidia mL^−1^ was beneficial to the parasitoid. The parasitoid used its initiative to inhibit the growth and reproduction of *B. bassiana*, ensuring its population reproduction. Interspecific relationships within cohorts are complex and diverse. Different species have various tactics that are not always fatal but could be more adaptable and versatile to preserve the interactions between the three species. In conclusion, and from the perspective of integrated pest management (IPM), our results show the negative interactions between the co-application of *S.guani* and *B. bassiana* due to the adverse effects provided by the entomopathogen on the survival and development of the parasitoids. However, our research also indicates that the application of these two biological control agents could potentially be used in combination to control *M. alternatus*, wherein this use requires effective time management to avoid antagonistic interactions.

In the experiment, we found an interesting phenomenon whereby, under the 10^6^ conidia mL^−1^, the parasitoids died on day 5 after inoculation and could not complete the whole growth and development. However, at 10^4^ conidia mL^−1^, this had little effect on parasitoids. Parasitoids can survive under 10^5^ conidia mL^−1^, although some individuals can also grow hypha and cause death, and most of the individuals survive. This may be because parents inhibit the growth of *B. bassiana* because of parental care [16]. Some insects release chemical information to inhibit organisms in the community that is not conducive to parasitoid development [38,39], while some of the individual bodies can grow hyphae. We inferred that in response to pathogenic fungal stress, the parent adult females of *S. guani* reduce their fertility and also indirectly affect the offspring’s survival and development. We have also verified through some experiments that the parent carrying a specific concentration will transmit the *B. bassiana* to his offspring [40]. The offspring can also carry the pathogenic fungus after emerging [40]. Therefore, we speculate that the parasitoids compete for speed between the two, first by inhibiting the growth and development of the entomopathogenic microorganisms and then shortening the time to subdue the host. Second, we question whether there is a parasite that preempts the growth rate, but *B. bassiana* growth is retarded rather than completely disappearing, such that the parasitoid’s offspring can be transferred to the subsequent infection cycle following emergence. Through this study, we can better explain the relationship between various organisms in the cogroup.

## Figures and Tables

**Table 1:** LE and PE of *S. guani* on the larvae of *M. alternatus* under different concentrations of *B. bassiana* spore suspensions.

Concentrations (conidia mL^−1^)	Parameters						LE	PE
	*D*(d)	*S*(d)	*t*(d)	*θ*	*P_R_*	*δ*	%	%
Control	6.67	3.67	3.00	1.8174	0.3549	0.8876	90.00	83.33
1 × 10^4^	5.93	3.44	2.49	1.7238	0.3671	0.8950	93.33	86.67
1 × 10^5^	5.17	3.20	1.97	1.6156	0.3771	0.9047	93.33	93.33
1 × 10^6^	5.00	3.10	1.90	1.6112	0.3892	0.9052	100.00	96.67

LE (lethal effect): the effect on survival; PE (parasitic effect): the average number of parasitic pests per natural enemy; *D*: the pre-oviposition of female adult parasitoids; *S*: the female adult parasitoids completely paralyze the host at the time; *t*: the female adult parasitoids die after the host due to the duration of egg laying; *θ*: the ratio of the time it takes for the female adult parasitoids to completely paralyze the host during pre-oviposition of the female adult parasitoids; *P_R_*: the parasitic probability of *S. guani* after paralyzing host *M. alternatus* larvae; *δ*: the antagonistic effect of *S. guani* and *B. bassiana*;δ: the antagonistic effect of *S. guani* and *B. bassiana* (the greater the antagonism, the worse the parasitoid, the weaker the lethal host effect).

**Figure A2:**
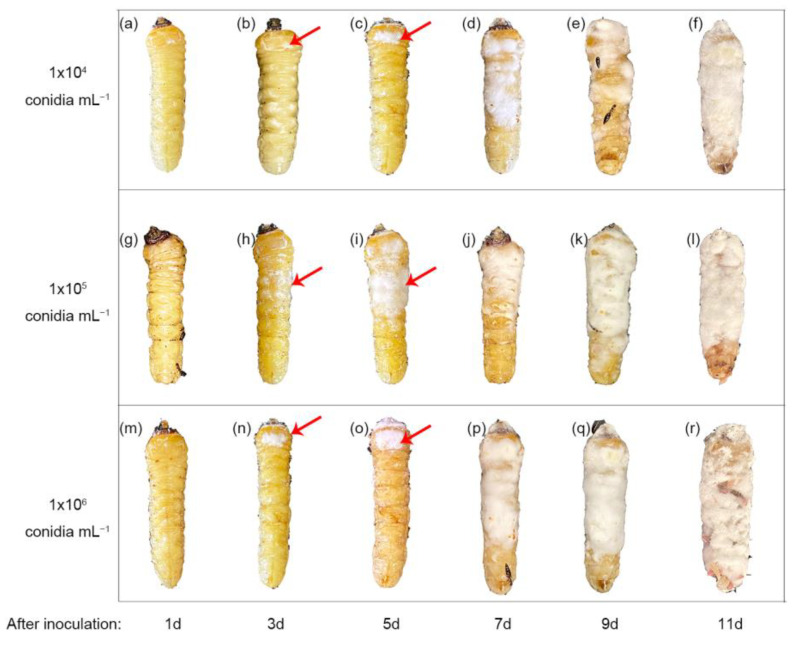
“*S. guani-B. bassiana-M. alternatus*” coexist in space, showing the hyphae of the *B. bassiana* grow rate. (a–f: growth of hyphae at 10^4^ conidia mL^−1^ *B. bassiana* suspension; g–l: growth of hyphae at 10^5^ conidia mL^−1^ *B. bassiana* suspension; m–r: growth of hyphae at 10^6^ conidia mL^−1^ *B. bassiana* suspension).

**Figure A3:**
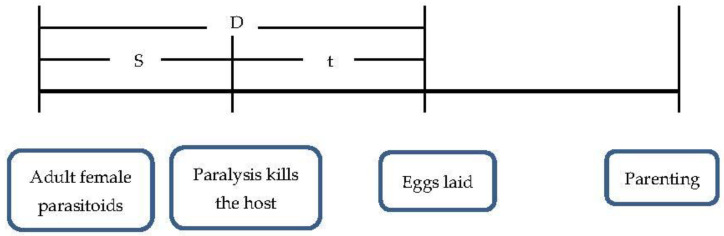
Exploded view of the model-the mortality effect of *S. guani* on the host under *B. bassiana* stress’s supplementary mate.

## Data Availability

The data are contained within the article.

## Appendix B

*D*: the pre-oviposition of female adult parasitoids;

*S*: the female adult parasitoids completely paralyze the host at the time;

*B*: the time when the female adult parasitoid delivers a fatal blow to the host;

*t*: the female adult parasitoids die after the host due to the duration of egg laying;

*θ*: the ratio of the time it takes for the female adult parasitoids to completely paralyze the host during pre-oviposition of the female adult parasitoids;

*P_R_*: the parasitic probability of *S. guani* after paralyzing host *M. alternatus* larvae;

*δ*: the antagonistic effect of *S. guani* and *B. bassiana*.
